# Predictive modeling for score translation among patient-reported outcome measures in chronic rhinosinusitis with nasal polyps: a cross-sectional study

**DOI:** 10.1007/s00405-025-09446-1

**Published:** 2025-06-18

**Authors:** J. M. García-Fernández, M. S. Sánchez-Torices, M. A. Feliz-Fernández, R. Lomas-Vega, M. A. Montilla-Ibáñez

**Affiliations:** 1 Present Address: Department of Health Sciences, University of Jaén, Jaén, Spain; 2 Department of Otolaryngology, Hospital Universitario de Jaén, Jaén, Spain

**Keywords:** Chronic rhinosinusitis, CRSwNP, PRO questionnaires, Predictive modeling, SNOT-22, NOSE, CRS-PRO, NPQ, Data harmonization, Clinical outcomes

## Abstract

**Background:**

Patient-reported outcome (PRO) questionnaires are essential tools for evaluating symptom burden and quality of life in patients with chronic rhinosinusitis with nasal polyps (CRSwNP). Instruments such as NOSE, SNOT-22, CRS-PRO, and NPQ are commonly used; however, the capability to translate scores between these instruments remains largely unexplored, limiting cross-study comparisons and continuity in patient care.

**Objective:**

To develop and validate predictive models quantifying relationships between widely utilized PRO questionnaires in CRSwNP and to assess their practical implications for clinical management and integration into research.

**Methods:**

In this observational cross-sectional study, 200 patients with CRSwNP completed the NOSE, SNOT-22, CRS-PRO, and NPQ questionnaires. Pairwise predictive models were constructed using linear and Random Forest regression methods. Model performance was evaluated through metrics such as R² and mean squared error (MSE). Model validity was ensured using the Durbin-Watson, Breusch-Pagan, Shapiro-Wilk, and variance inflation factor (VIF) tests. Clinical subgroup analyses based on variables such as asthma and prior nasal surgery were also conducted.

**Results:**

Strong correlations among questionnaires were observed (r=0.61–0.87). Linear regression models demonstrated high predictive accuracy, notably for SNOT-22 predicting NPQ (R²=0.76), NPQ predicting CRS-PRO (R²=0.76), and CRS-PRO predicting SNOT-22 (R²=0.74). Random Forest models showed minor performance enhancements (ΔR²≤0.03). Subgroup analyses indicated increased predictive precision in patients with asthma or previous nasal surgery. These predictive models enable clinicians to interpret scores across different instruments confidently, optimizing patient management decisions, particularly in monitoring treatment responses and longitudinal follow-ups.

**Results:**

Predictive modeling among PRO questionnaires in CRSwNP is both feasible and clinically impactful. These models facilitate the translation of scores between instruments, thus enhancing clinical decision-making, streamlining patient assessments, and supporting data harmonization in multicentric and longitudinal studies. Future research should pursue external and longitudinal validations to ensure broader applicability and reliability of these predictive tools.

## Introduction

Chronic rhinosinusitis (CRS) is a prevalent inflammatory condition affecting approximately 10–12% of the population in Europe and North America, posing significant burdens on both individuals and healthcare systems [1]. Among CRS subtypes, chronic rhinosinusitis with nasal polyps (CRSwNP) is particularly notable due to its increased severity and chronicity, presenting symptoms such as persistent nasal obstruction, rhinorrhea, anosmia, and facial pain or pressure [2]. Moreover, CRSwNP often coexists with comorbid conditions such as asthma and aspirin-exacerbated respiratory disease (AERD), further complicating its clinical management and adversely affecting patients’ quality of life [2, 3].

In the clinical assessment and management of CRSwNP, patient-reported outcome (PRO) measures have emerged as indispensable tools, providing insights into symptom burden, psychosocial impacts, and overall quality of life from the patient’s perspective. However, the multiplicity of PRO instruments currently in use presents challenges for both research and clinical practice, particularly when attempting to compare outcomes across different studies or integrate data from various clinical centers utilizing diverse assessment tools.

Several widely validated PRO questionnaires are commonly employed in CRSwNP evaluation. The Nasal Obstruction Symptom Evaluation (NOSE) questionnaire, validated in 2004, succinctly captures patients’ perceptions of nasal blockage [2]. In contrast, the Sinonasal Outcome Test-22 (SNOT-22), introduced in 2009, provides a broader and comprehensive assessment of sinonasal symptoms and their impact on quality of life [4]. Recently developed instruments such as the Chronic Rhinosinusitis Patient-Reported Outcome (CRS-PRO), validated in 2020, integrate both physical and psychosocial dimensions, offering a holistic measure of disease burden [5]. Similarly, the Nasal Polyposis Quality of Life (NPQ), published in 2022, is specifically tailored to evaluate quality of life in CRSwNP patients [6].

Although each of these questionnaires has been individually validated and has proven clinical utility, the statistical and clinical interrelationships between them remain insufficiently explored. Understanding these relationships could significantly enhance clinical and research practices by enabling the translation of scores across instruments, thus facilitating longitudinal tracking of disease progression, comparisons between different patient cohorts, and harmonization of data in multi-center or longitudinal studies [7]. Additionally, predictive modeling between these PRO measures could alleviate patient burden by permitting clinicians and researchers to choose context-appropriate instruments without compromising comparability and continuity of patient data.

To date, no comprehensive analysis has systematically addressed the predictive relationships among these commonly used PRO instruments in CRSwNP. The current study aims to fill this gap by developing and validating robust predictive models among the NOSE, SNOT-22, CRS-PRO, and NPQ questionnaires. By elucidating the statistical interdependence of these instruments, this research seeks to establish a solid foundation for the integrated application of PRO measures, thus promoting consistency, minimizing redundancy, and ultimately enhancing patient-centered care in CRSwNP.

## Materials and methods

This observational cross-sectional study was designed to evaluate predictive relationships among widely utilized PRO questionnaires in patients diagnosed with CRSwNP. The study was conducted at the Rhinology Clinic of University Hospital of Jaén, a tertiary care center. A consecutive sample of 200 adult patients aged 18 years and older, who met diagnostic criteria for bilateral nasal polyps as per the European Position Paper on Rhinosinusitis and Nasal Polyps (EPOS) 2020 guidelines, were enrolled. Patients were excluded if they presented with central nervous system disorders, malignancies, acute infections, previous head and neck radiotherapy, or significant unrelated comorbidities that could impact questionnaire responses.

Participants completed four validated PRO questionnaires: NOSE, SNOT-22, CRS-PRO, and NPQ, during a single clinical visit. Additional demographic and clinical data, including the presence of asthma, allergies, sleep apnea, prior nasal surgery, and surgical indications, were recorded to enable subgroup analyses aimed at exploring whether specific clinical characteristics influence predictive model performance.

Initial descriptive statistics including mean, median, standard deviation, and interquartile range (IQR) were computed for each PRO measure. Relationships among questionnaires were preliminarily assessed using Pearson’s correlation coefficients and visualized via heatmaps.

Predictive modeling involved pairwise comparisons of questionnaires, using both linear regression and Random Forest regression approaches. Linear regression models were primarily employed due to their interpretability, simplicity, and wide clinical acceptance. Random Forest models were concurrently evaluated to explore whether more complex, non-linear interactions could offer improved predictive accuracy, especially in cases of heteroscedasticity or non-linear relationships. Hyperparameter optimization, including number of trees and maximum depth, was performed through grid search combined with cross-validation using an 80 − 20 train-test data split.

Model performance was rigorously validated through established statistical metrics including the coefficient of determination (R²) and mean squared error (MSE). Additionally, residual diagnostics were conducted to ensure adherence to statistical assumptions. These included the Durbin-Watson statistic to assess error independence, Breusch-Pagan tests for homoscedasticity, Shapiro-Wilk tests for normality, and evaluation of variance inflation factors (VIF) to rule out multicollinearity.

Subgroup analyses were also conducted, stratifying patients by clinical variables such as asthma status, history of nasal surgery, allergy, sleep apnea, and surgical indication. Separate regression models were developed within these subgroups to determine if and how clinical heterogeneity influences predictive accuracy and the robustness of the questionnaire relationships. All statistical analyses were performed using Python version 3.9, specifically utilizing Scikit-Learn, Statsmodels, Matplotlib, and Seaborn libraries for data processing and visualization.

## Ethical approval and informed consent

This study was conducted in accordance with the ethical principles outlined in the Declaration of Helsinki and was approved by the Research Ethics Committee of [Hospital Universitario de Jaén] (Approval Code: SICEIA-2024-001376). All participants provided written informed consent prior to their inclusion in the study. The confidentiality of patient data was maintained in compliance with institutional and international ethical standards.

## Results

### General characteristics of participants

The study included a total of 200 consecutive patients diagnosed with chronic rhinosinusitis with nasal polyps (CRSwNP). The mean age of the participants was 48 ± 12 years, with an age range of 19 to 73 years, and 58% of them were male. The average scores were as follows: NOSE (mean = 12 ± 5), SNOT-22 (mean = 45 ± 18), CRS-PRO (mean = 22 ± 8), and NPQ (mean = 33 ± 10). Distribution analyses showed that most variables approached normality, although SNOT-22 and NPQ scores showed slight right-skewed tendencies, reflecting a concentration of patients with higher symptom burden and quality-of-life impact.

Analysis of the score distributions using histograms revealed that, although most of the questionnaires exhibited normal distributions, the SNOT-22 and NPQ demonstrated slight skewness towards higher values.

### Relationships between questionnaires

**Fig. 1:**
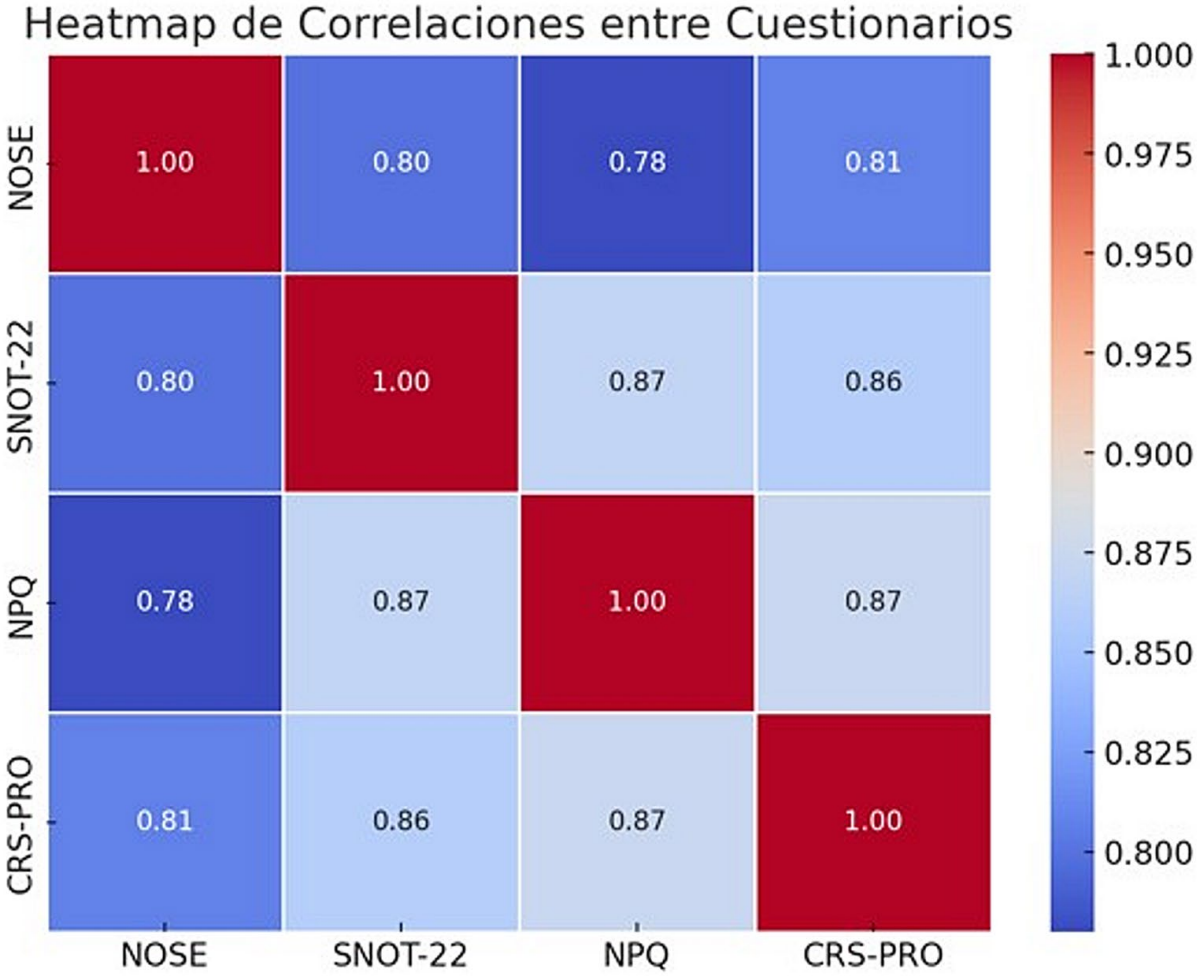
Heatmap of the correlation matrix among the NOSE, SNOT-22, CRS-PRO, and NPQ questionnaires. Darker shades represent stronger correlations (*r* > 0.7)

The correlations between the questionnaires were strong and statistically significant, highlighting the interdependence of these instruments in measuring aspects of quality of life and symptoms in patients with CRSwNP. Pearson’s correlation coefficients ranged from 0.61 (NOSE–NPQ) to 0.87 (SNOT-22–NPQ and NPQ–CRS-PRO), all with p-values < 0.001. These findings confirm the interdependence of these tools in assessing the multifaceted symptomatology and impact of CRSwNP. These relationships are illustrated in Fig. 1, which presents the heatmap of the correlation matrix among the NOSE, SNOT-22, CRS-PRO, and NPQ questionnaires.

### Predictive models

Linear regression models demonstrated high predictive accuracy for most questionnaire pairings. Notably, SNOT-22 emerged as a highly informative predictor for both NPQ (R² = 0.76, MSE = 81.69) and CRS-PRO (R² = 0.74, MSE = 24.72), while NPQ was similarly effective in predicting SNOT-22 (R² = 0.76) and CRS-PRO (R² = 0.76). CRS-PRO also proved to be a strong predictor of NPQ (R² = 0.76). These models suggest a meaningful overlap between domains of general symptom burden, psychosocial impact, and nasal-specific quality-of-life indicators.

On the other hand, predictions involving the NOSE questionnaire showed slightly lower R² values, particularly in predicting NPQ (R² = 0.61). This likely reflects the focused symptom scope of the NOSE, which is limited to nasal obstruction and may not fully capture broader dimensions such as fatigue, emotional distress, or social limitations addressed in NPQ and SNOT-22.

All models were statistically significant with p-values < 0.001. A summary of model performance is presented in Table 1.

### Random forest comparison

To assess potential improvements in predictive accuracy, Random Forest models were applied to the same pairings. While Random Forest slightly outperformed linear regression in some cases—such as SNOT-22 → NPQ (R² increased from 0.76 to 0.78) and CRS-PRO → SNOT-22 (R² from 0.74 to 0.77)—the gains were modest. In most cases, Random Forest yielded comparable R² and MSE values to linear regression, suggesting that the added model complexity did not result in clinically meaningful improvement.

These results indicate that linear models are sufficiently robust for practical use, particularly considering their interpretability in clinical settings.

These findings highlight that Random Forest does not drastically outperform traditional linear regression for PRO questionnaire modeling in CRSwNP patients. However, its ability to model complex, non-linear interactions warrants further exploration in future studies (Table 2).

**Table 1:** Linear regression models

Predictor	Target	*R*^2	MSE	*P*-value (<0.05)
NOSE	SNOT-22	0.64	117.28	Yes
NOSE	NPQ	0.61	131.2	Yes
NOSE	CRS-PRO	0.65	33.16	Yes
SNOT-22	NOSE	0.64	6.59	Yes
SNOT-22	NPQ	0.76	81.69	Yes
SNOT-22	CRS-PRO	0.74	24.72	Yes
NPQ	NOSE	0.61	7.15	Yes
NPQ	SNOT-22	0.76	79.32	Yes
NPQ	CRS-PRO	0.76	22.59	Yes
CRS-PRO	NOSE	0.65	6.33	Yes
CRS-PRO	SNOT-22	0.74	84.05	Yes
CRS-PRO	NPQ	0.76	79.09	Yes

**Table 2:** Predictive model equations

Predictor	Target	Equation
NOSE	SNOT-22	SNOT-22 = 7.66 + 3.37 * NOSE
NOSE	NPQ	NPQ = -0.01 + 3.34 * NOSE
NOSE	CRS-PRO	CRS-PRO = 1.34 + 1.85 * NOSE
SNOT-22	NOSE	NOSE = 1.91 + 0.19 * SNOT-22
SNOT-22	NPQ	NPQ = -3.4 + 0.88 * SNOT-22
SNOT-22	CRS-PRO	CRS-PRO = 0.32 + 0.47 * SNOT-22
NPQ	NOSE	NOSE = 3.65 + 0.18 * NPQ
NPQ	SNOT-22	SNOT-22 = 12.44 + 0.86 * NPQ
NPQ	CRS-PRO	CRS-PRO = 4.05 + 0.47 * NPQ
CRS-PRO	NOSE	NOSE = 2.75 + 0.35 * CRS-PRO
CRS-PRO	SNOT-22	SNOT-22 = 9.59 + 1.59 * CRS-PRO
CRS-PRO	NPQ	NPQ = 0.72 + 1.64 * CRS-PRO

### Model validation

All regression models were subjected to diagnostic testing. Durbin-Watson values ranged between 1.85 and 2.17, supporting independence of residuals. The Breusch-Pagan test revealed acceptable homoscedasticity in most models, although mild heteroscedasticity was observed in pairings involving NOSE and NPQ. Shapiro-Wilk tests showed that residuals were not normally distributed in most models; however, residual plots confirmed acceptable behavior for linear modeling. Multicollinearity was not present in any models, with all VIF values ≤ 1.0 (Table 3).

**Table 3:** Validation of model assumptions

Predictor	Target	Durbin-Watson	Breusch-Pagan *p*-value	Shapiro-Wilk *p*-value	VIF
NOSE	SNOT-22	2.04	0.0068	0.0	1.0
NOSE	NPQ	2.08	0.0001	0.0	1.0
NOSE	CRS-PRO	2.1	0.0018	0.0	1.0
SNOT-22	NOSE	2.15	0.1539	0.0	1.0
SNOT-22	NPQ	1.91	0.0008	0.0	1.0
SNOT-22	CRS-PRO	1.99	0.8288	0.0	1.0
NPQ	NOSE	2.13	0.4961	0.0	1.0
NPQ	SNOT-22	1.85	0.726	0.0001	1.0
NPQ	CRS-PRO	1.92	0.8494	0.0002	1.0
CRS-PRO	NOSE	2.17	0.0036	0.0	1.0
CRS-PRO	SNOT-22	1.95	0.2836	0.0	1.0
CRS-PRO	NPQ	1.94	0.0	0.0002	1.0

### Subgroup performance and clinical heterogeneity

To further explore the robustness of the predictive models, we conducted stratified analyses across clinically relevant subgroups, including asthma, sleep apnea, allergy, prior nasal surgery, and surgical indication. Results revealed notable differences in model performance based on clinical characteristics.

In patients with asthma, predictive accuracy was consistently higher across most models. For example, the NOSE → CRS-PRO model showed an R² of 0.69 in asthmatic patients compared to 0.58 in non-asthmatics, suggesting greater consistency between nasal obstruction and global symptom burden in this subgroup.

Similarly, in those with prior nasal surgery, the CRS-PRO → NPQ model achieved an R² of 0.81, outperforming the same model in surgery-naïve patients (R² = 0.74). This may reflect a higher alignment between quality-of-life measures in patients with a longer disease course or surgical history.

Models stratified by presence of allergy or sleep apnea showed less variability in performance, indicating more stable inter-questionnaire relationships across these subgroups.

These findings support the idea that clinical characteristics influence how symptom domains relate to one another, and that predictive modeling may offer enhanced value in specific subgroups—particularly in those with more complex or severe disease phenotypes.

## Discussion

### Predictive relationships between questionnaires in chronic rhinosinusitis with nasal polyps

This study is the first to systematically model the predictive relationships among four validated patient-reported outcome (PRO) questionnaires in chronic rhinosinusitis with nasal polyps (CRSwNP): the NOSE, SNOT-22, CRS-PRO, and NPQ. Our results show strong correlations and significant predictive accuracy between instruments, particularly between SNOT-22, NPQ, and CRS-PRO. These findings not only highlight the conceptual overlap between these tools but also support the feasibility of score translation across questionnaires through statistical modeling.

### Clinical interpretation and utility

The key contribution of this study lies in its clinical utility. In routine practice, the choice of PRO instrument often depends on institutional preference, resource availability, or the specific context (e.g., preoperative vs. postoperative monitoring, biologic therapy evaluation). Our findings suggest that clinicians can confidently extrapolate scores from one questionnaire to another using the proposed models—particularly in cases where only one instrument is routinely used.

For example, in settings where only the NOSE is used (e.g., surgical clinics focused on nasal obstruction), our models allow clinicians to estimate a patient’s expected CRS-PRO or SNOT-22 score, thereby gaining insights into psychosocial burden or global symptom impact. Similarly, if longitudinal data collection shifts from NPQ to SNOT-22 (or vice versa), our models can help harmonize data across timepoints without introducing measurement bias.

Although some predictive relationships were less robust (e.g., NOSE → NPQ, R² = 0.61), these limitations likely reflect differences in questionnaire scope. The NOSE is focused exclusively on obstruction, while the NPQ encompasses broader quality-of-life domains. In such cases, our findings emphasize the need for comprehensive tools when assessing disease burden in full.

### Comparison with previous literature

Several previous studies have emphasized the utility of patient-reported outcome (PRO) questionnaires in assessing the impact and treatment response of chronic rhinosinusitis (CRS). However, the development of predictive models that integrate multiple PRO instruments remains a relatively underexplored area. Our findings contribute to this evolving field by demonstrating that questionnaires targeting distinct but related symptom domains—such as nasal obstruction (NOSE), overall symptom burden (SNOT-22), and quality of life (NPQ, CRS-PRO)—can be statistically linked with high predictive accuracy [8, 9].

This multidimensional integration aligns with earlier work advocating for composite assessment approaches in CRS to capture the full breadth of disease burden [8, 10]. For example, it has been suggested that tools like the SNOT-22 and CRS-PRO complement each other in evaluating both physical symptoms and psychosocial impact [9, 11]. Our models not only support this conceptual overlap but also offer a practical means of translating scores, which could facilitate data harmonization across studies and clinical settings [10, 12].

Furthermore, the application of predictive modeling enhances the clinical relevance of these instruments by allowing flexibility in questionnaire selection without compromising the richness of the information obtained. This is particularly relevant in the context of biologic therapies, where CRS-PRO has been proposed as a tool to monitor treatment response. The strong predictive performance observed in models such as CRS-PRO → SNOT-22 (R² = 0.74) reinforces its potential in longitudinal monitoring, even when direct measurement with multiple instruments is not feasible [11, 13].

Although we explored Random Forest models to assess potential nonlinearities in symptom relationships, improvements over traditional linear regression were modest. Given their interpretability and ease of clinical implementation, linear models remain the preferred approach for score translation in practice [14].

Ultimately, this study extends the existing literature by demonstrating that predictive equivalence across PRO instruments is both feasible and clinically valuable, offering a framework to enhance continuity, reduce redundancy, and improve outcome tracking in CRSwNP management [15, 16].

These findings suggest that while linear regression remains a valid approach for modeling relationships among PRO questionnaires, the use of non-linear models such as Random Forest may be beneficial in scenarios where symptom interactions and quality-of-life measures exhibit greater complexity. The application of machine learning techniques in PRO research represents a promising avenue for future studies, particularly in refining cross-study comparisons and harmonizing patient-reported data.

### Limitations and future perspectives

This study has several limitations. First, its cross-sectional design does not permit evaluation of how inter-questionnaire relationships evolve over time or in response to treatment interventions. Second, the data were obtained from a single tertiary care center, which may limit the generalizability of the findings to other healthcare settings or broader populations. Third, while subgroup analyses were performed based on key clinical variables—such as asthma, allergy, sleep apnea, and prior nasal surgery—the study was not powered to detect subtle differences across all potential subpopulations. Therefore, these exploratory findings should be interpreted with caution and validated in larger, multicenter cohorts. Additionally, the absence of longitudinal follow-up precludes assessment of model stability over time.

### Future directions

Future studies should validate these models in multicenter and longitudinal settings. External validation would confirm the robustness of predictive performance across populations, while repeated-measures designs could assess whether score translation remains stable over time or under treatment pressure (e.g., after biologic therapy). Moreover, machine learning methods should continue to be explored, particularly in tailoring predictions to clinical subgroups or creating adaptive PRO selection tools based on patient characteristics.

## Conclusion

The present study confirms that predictive modeling among commonly employed patient-reported outcome (PRO) instruments in chronic rhinosinusitis with nasal polyps (CRSwNP) is both methodologically sound and clinically meaningful. The high degree of correlation and strong predictive performance—particularly among the SNOT-22, CRS-PRO, and NPQ—underscores the potential for score translation across instruments in appropriate clinical scenarios.

From a pragmatic standpoint, these models represent a valuable resource for clinicians and investigators seeking to streamline PRO usage. They facilitate data harmonization in multicenter and longitudinal studies, minimize patient burden by reducing redundant assessments, and allow tailored instrument selection based on clinical priorities—be it brevity, domain specificity, or comprehensiveness. Importantly, they offer a feasible strategy to maintain longitudinal continuity when different tools are used across timepoints or settings.

Although these models are not intended to substitute for comprehensive assessments when required, they provide a practical framework for enhancing consistency in outcome reporting. Their relevance is especially pronounced in the era of biologic therapies, where the ability to capture and compare multi-domain symptom trajectories is essential, yet often challenged by heterogeneous PRO use.

Future studies should aim to externally validate these models across diverse populations and over time. Nonetheless, this work represents an important step toward a more integrated, flexible, and patient-centered application of PROs in the management of CRSwNP.
